# Impact and management of integrated dysphagia rehabilitation within cardiac care programs for older patients with cardiovascular disease

**DOI:** 10.1007/s41999-024-01031-9

**Published:** 2024-08-09

**Authors:** Hiroaki Obata, Tohru Izumi, Mitsuo Ishizuka, Kenji Yamaguchi, Naohito Hao, Nobue Yagihara, Satoru Abe, Hiroshi Watanabe, Takayuki Inomata, Shigeru Makita, Shigeru Fujimoto

**Affiliations:** 1 Division of Internal Medicine, Niigata Minami Hospital, Niigata, Japan; 2 Division of Rehabilitation, Niigata Minami Hospital, Niigata, Japan; 3 Department of Cardiovascular Medicine, Niigata University Graduate School of Medical and Dental Sciences, 757 Ichiban-cho, Asahimachi-dori, Chuo-ku, Niigata, 950-8510 Japan; 4 Laboratory on Internal Disability Rehabilitation for Community Health Networks, Niigata University Graduate School of Medical and Dental Sciences, Niigata, Japan; 5 Division of Dentistry, Niigata Minami Hospital, Niigata, Japan; 6 Department of Rehabilitation Medicine, Saitama Medical University International Medical Center, Saitama, Japan; 7 Department of Rehabilitation Medicine, Kawaguchi Cupola Rehabilitation Hospital, Saitama, Japan; 8 Division of Neurology, Department of Medicine, Jichi Medical University, Tochigi, Japan

**Keywords:** Dysphagia rehabilitation, Older patients, CVD, Cardiac rehabilitation, Super-aged society

## Abstract

**Purpose:**

This study assessed the characteristics, management, and outcomes of dysphagia rehabilitation in older patients with CVD in a super-aged society, highlighting the need for comprehensive management strategies in community hospital settings. It aimed to uncover valuable insights into the benefits of integrating dysphagia rehabilitation with cardiac care in patient management.

**Methods:**

We conducted a retrospective review of patients with CVD aged ≥ 65 years who were admitted to Niigata Minami Hospital between January 2019 and December 2021. We focused on patients requiring dysphagia rehabilitation and assessing the effects of these interventions on recovery.

**Results:**

The study included 732 participants with an average age of 86.0 ± 7.8 years, of whom 41.9% were male. Approximately 55.1% required dysphagia rehabilitation. Dysphagia rehabilitation significantly improved oral caloric intake and BMI in patients who underwent rehabilitation, and these improvements were comparable to those in patients who did not require dysphagia rehabilitation. Significant enhancement in the ADL of patients was observed at discharge. Patients who required dysphagia rehabilitation also had longer hospital stays and were more likely to be discharged to nursing facilities.

**Conclusion:**

Dysphagia is common in older patients with CVD, and dysphagia rehabilitation positively affects the maintenance of nutritional status and helps patients achieve ADL independence at discharge. This study highlights the importance of integrating dysphagia rehabilitation into ordinary cardiac rehabilitation programs for older patients with CVD to improve their QOL.

## Key summary points

**Aim:**

To explore the characteristics and management of dysphagia among older hospitalized patients with cardiovascular disease (CVD) in Japan and to clarify the impact of integrated dysphagia rehabilitation on oral intake, body mass index (BMI), and activities of daily living (ADL).

**Findings:**

Dysphagia is prevalent among older patients with CVD and significantly impacts their recovery. Integrated dysphagia rehabilitation can improve oral intake, BMI, and ADL independence.

**Message:**

Active integration of dysphagia management into cardiac care programs is crucial for enhancing the overall recovery and QOL in older patients with CVD.

## Introduction

The prevalence of CVD in older adults has escalated to become a major global health concern as society continues to age [1]. These patients frequently exhibit various comorbidities, including dysphagia, characterized by difficulty in swallowing, that significantly impair their quality of life (QOL) by increasing the risk of malnutrition and aspiration pneumonia. Despite its acknowledged clinical relevance in older patients with CVD [2, 3], the integration of dysphagia management and rehabilitation within cardiac care programs is often overlooked and only sporadically implemented.

This study aims to explore the prevalence and management of dysphagia among older hospitalized patients with CVD in Japan, which is home to the world’s largest aged population, and to clarify the impact of integrated dysphagia rehabilitation on oral intake, BMI, and ADL, thereby underscoring its potential to enhance the overall recovery and QOL in these patients. In addition, it sought to uncover valuable insights into the comprehensive benefits of integrating dysphagia rehabilitation with cardiac care in the patient management process.

## Methods

### Ethical considerations

This study was conducted using existing clinical data without patient samples. The study protocol was approved by the Ethics Review Committee of Niigata University (Approval Number: 2022–0118) and the Ethics Committee of Niigata Minami Hospital (Approval Number: 1909). In accordance with the ethical guidelines for medical and health research involving human subjects, the participants were informed about the study through posters displayed within the facility and information posted on the Niigata Minami Hospital website, based on the opt-out consent form provided by the Niigata University Research Ethics Committee. Participants had the option to opt out of the study and those who chose to do so were excluded.

### Study population

This retrospective study included patients ≥ 65 years who were admitted to the Niigata Minami Hospital from January 1, 2019, to December 31, 2021, with CVDs as the primary reason for their admission. Eligibility was determined using the diagnostic procedure combination (DPC) records, which included data such as primary diagnosis, reason for admission, or diagnosis requiring the most medical resources. Patients who were undergoing or eligible for rehabilitation interventions specifically prescribed based on clinical requirements during their hospital stay were included in this study. Data were retrospectively extracted from electronic medical records and anonymized for analysis.

### Setting

Niigata Minami Hospital is located in the urban area of Niigata, a regional city. The facility comprises 108 acute care beds, 35 community-based care beds, and 34 comprehensive rehabilitation beds, totaling 177 beds. Although the hospital does not have an intensive care unit, specialized cardiovascular care beds, or catheterization facilities, it is staffed with cardiovascular specialists, registered instructors in cardiac rehabilitation, and certified heart failure educators. As a community-based hospital, it responds to a wide range of local healthcare needs from emergency services to home visits. The hospital is particularly focused on accepting transferred patients for post-acute rehabilitation or discharge support from neighboring high-acuity hospitals that specialize in cardiac surgery and catheterization. This makes the hospital an ideal location for studying the rehabilitation needs and outcomes of older patients with CVDs.

### Study variables

We investigated several clinical background factors including age at admission, sex, BMI, primary diseases, comorbidities, left ventricular ejection fraction (EF) on echocardiography, and brain natriuretic peptide (BNP) levels. Diseases were analyzed using the International Classification of Diseases, Tenth Revision codes, derived from the DPC diagnoses, and laboratory data were extracted from tests conducted within five days of admission, prioritizing the results closest to the admission date. In addition, data on the length of hospital stay, living situation prior to admission, and number of days and average daily time (minutes) of physical and dysphagia rehabilitation were collected from medical records. The Barthel Index (BI) was used as a measure of the ADL, with pre-illness BI assessed through standardized questionnaires filled out by patients or their primary caregivers, and admission and discharge BI collected from the DPC data. Ganpule’s estimation formula, which is considered suitable for older Asians [4, 5], was used to estimate the basal metabolic rate of oral intake.

### Diagnosis and rehabilitation of dysphagia

**Fig. 1:**
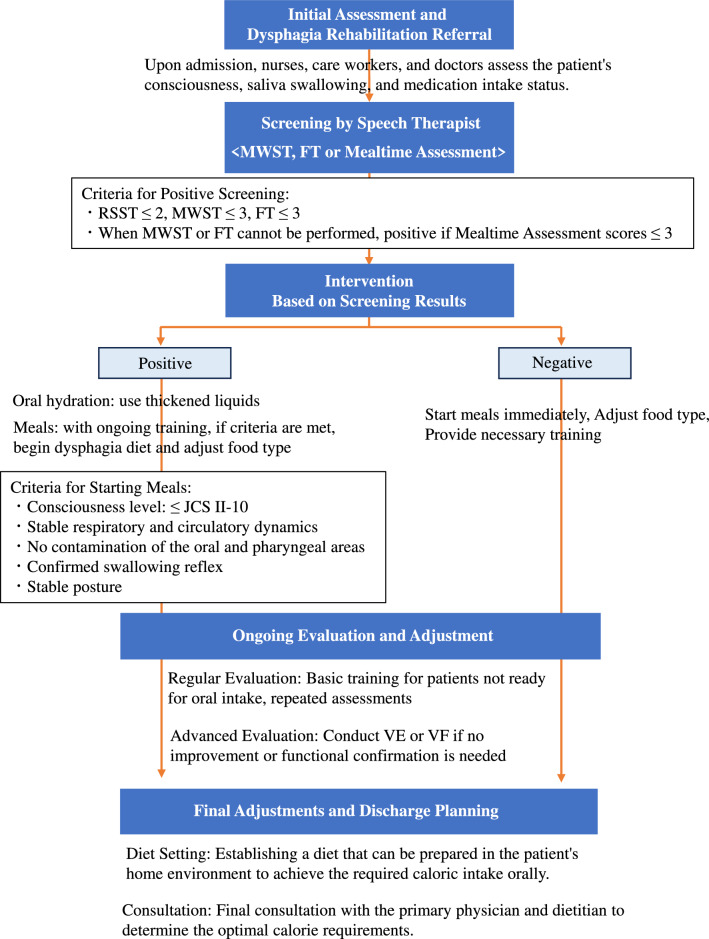
Workflow for dysphagia rehabilitation in hospitalized patients. Screening procedures include the Modified Water Swallow Test (MWST), Food Test (FT), and, when feasible, the Repetitive Saliva Swallowing Test (RSST). Positive results are defined as RSST ≤ 2, MWST ≤ 3, and FT ≤ 3. Patients identified as positive are advised on the use of thickened liquids and undergo training with adjusted food types. The Japan Coma Scale (JCS) is used to assess consciousness levels, with JCS II-10 indicating a level where arousal is easily achieved with normal stimuli. Patients identified as negative can immediately begin meals with necessary training. Repeated assessments, VE, and VF are conducted if no improvement is observed. The goal is to establish a manageable diet and achieve necessary calorie intake in collaboration with the primary physician and dietitian. *VE* videoendoscopic evaluation of swallowing, *VF* videofluoroscopic examination of swallowing

When there are concerns about oral intake or a need for rehabilitation, nurses, caregivers, and attending physicians consider evaluations from previous medical institutions, if available, and refer the patient to a speech therapist (ST). The referred ST conducts screening tests, including the Modified Water Swallow Test (MWST) and Food Test (FT). The Repetitive Saliva Swallowing Test (RSST) is also performed when feasible, though its implementation is limited among older patients because of cognitive impairments and other medical conditions. As a result, the MWST and FT are more commonly used to determine dysphagia. Positive (abnormal) results are defined as RSST ≤ 2, MWST ≤ 3, and FT ≤ 3 (Fig. 1).

Patients who are instructed to fast owing to medical conditions, those for whom MWST or FT are deemed risky, and those who have difficulty understanding the protocol are evaluated during mealtime. If these patients are considered to score 3 or below on any of these tests during mealtime evaluation, they are classified as having positive screening results. Patients identified as positive are advised on the use of thickened liquids. Upon meeting criteria outlined in Fig. 1, oral intake is initiated with adjustments to food types while conducting training. Patients with negative screening results can immediately begin meals, with adjustments to food types and necessary training.

All patients undergo repeated assessments to evaluate oral intake capacity and receive basic training. If no improvement is observed during treatment or if functional confirmation is necessary, videoendoscopic evaluation of swallowing (VE) or videofluoroscopic examination of swallowing (VF) is conducted, although these procedures are often not feasible in patients with cognitive impairments or those for whom the procedures pose significant risks. The ultimate goal is to establish a diet manageable in the patient’s living environment and achieve necessary calorie intake orally in collaboration with the primary physician and dietitian.

### Integrated dysphagia rehabilitation within the cardiac care framework

The care for heart disease was based on comprehensive cardiac rehabilitation. This rehabilitation followed national guidelines for cardiac rehabilitation [6], which includes risk management during exercise and intensity setting, and was conducted by a multidisciplinary team composed of physical therapists, occupational therapists, physicians, nurses, dietitians, pharmacists, and social workers. At Niigata Minami Hospital, dysphagia rehabilitation and its management were seamlessly integrated into the cardiac care framework and adhered to insurance medical practices. Dysphagia rehabilitation employed standardized assessment and intervention methods [7], and the workflow was as outlined in the previous section. The team of speech-language therapists and dental hygienists was led by staff with over 10 years of experience, and the supervising dentist, who held a degree in dysphagia research, was responsible for conducting VE and VF and participating in diagnosis and treatment planning. The cardiac rehabilitation team and the dysphagia team worked closely with ward nurses and rehabilitation therapists, regularly exchanging information during case conferences to determine both discharge and post-discharge policies. Furthermore, for physical rehabilitation interventions, consultations were conducted with the attending physician, rehabilitation specialists, orthopedic surgeons, and neurologists.

### Statistical analysis

The patients were divided into two groups based on whether they underwent dysphagia rehabilitation or not. Continuous variables were reported as mean ± standard deviation or median (interquartile range), and categorical variables were presented as counts (%). Categorical variables were analyzed using the χ^2^ test where applicable, and the Fisher’s exact test was used otherwise. Temporal changes in the BI were assessed using the Wilcoxon rank-sum test, and differences between disease subgroups were evaluated using the Kruskal–Wallis rank sum test. Multivariate logistic regression analysis was conducted to identify factors associated with achieving independence in ADL at discharge. This analysis aimed to evaluate the impact of various predictors, including demographic characteristics, clinical factors, and the provision of dysphagia rehabilitation, on the likelihood of patients being independent at discharge, as measured using the BI. Statistical analyses were conducted using SPSS version 23.0 and EZR version 1.61. Statistical significance was set at *p* < 0.05.

## Results

The study enrolled 732 participants, predominantly older, with an average age of 86.0 ± 7.8 years; 307 (41.9%) were male. First, to clarify the characteristics of patients requiring dysphagia rehabilitation, we divided all cases into those requiring dysphagia rehabilitation (DR group) and those not requiring it (NDR group), as summarized in Table 1. Of these, 403 (55.1%) required dysphagia rehabilitation.

**Table 1:** Clinical background of the study participants

	DR group (*N* = 403)	NDR group (*N* = 329)	*p*-value
Age, years	87.1 ± 7.6	84.7 ± 7.9	< 0.01
Male	167 (41.4%)	140 (42.6%)	0.76
Underlying cardiac disease			
Ischemic heart disease	89 (22.1%)	75 (22.8%)	0.82
Valvular heart disease	65 (16.1%)	48 (14.6%)	0.57
Cardiomyopathy	11 (2.7%)	14 (4.3%)	0.26
Atrial fibrillation	189 (46.9%)	167 (50.8%)	0.55
Comorbidities			
Hypertension	246 (61.0%)	218 (66.3%)	0.15
Dyslipidemia	77 (19.1%)	108 (32.8%)	< 0.01
Diabetes mellitus	35 (8.7%)	38 (11.6%)	0.20
Hyperuricemia	51 (12.7%)	59 (17.9%)	0.05
CKD	80 (19.9%)	67 (20.4%)	0.86
Cognitive disorder	112 (27.8%)	69 (21.0%)	0.03
Rehabilitation-related Comorbid Conditions			
Chronic respiratory disease	74 (18.4%)	65 (19.8%)	0.63
Skeletal disease	61 (15.1%)	71 (21.6%)	0.02
Neurological disease	223 (55.3%)	156 (47.4%)	0.03
Disuse syndrome	207 (51.4%)	146 (44.4%)	0.06
Cancer	43 (10.7%)	20 (6.1%)	0.03
Residence before admission			
Own home	275 (68.2%)	236 (71.7%)	< 0.01
Nursing home	52 (12.9%)	19 (5.8%)	
Hospital (patient transfer)	76 (18.9%)	74 (22.5%)	
Length of hospital stay, days	47.3 ± 28.1	40.1 ± 25.6	< 0.01
≥ 30 days	279 (69.2%)	195 (59.3%)	< 0.01
In-hospital death	69 (17.1%)	25 (7.6%)	< 0.01
Assessment of Swallowing Function			
Days from admission to assessment	2.4 ± 5.9		
Positive MWST (%)	168 (41.7%)		
Positive FT (%)	79 (19.6%)		
Positve MWST and FT (%)	64 (15.9%)		
Positive MWST or FT (%)	183 (45.4%)		
VE performed (%)	43 (10.7%)		
VF performed (%)	14 (3.5%)		
Implementation of dysphagia rehabilitation			
Number of days	16.7 ± 18.7		
Average daily time, min/day	35.0 ± 9.8		
Implementation of physical rehabilitation			
Number of days	31.5 ± 22.6	30.9 ± 24.0	0.72
Average daily time, min/day	61.8 ± 27.4	75.9 ± 29.2	< 0.01

Variables are presented as mean ± standard deviation for continuous data and count (percentage) for categorical data. The rehabilitation-related comorbid conditions are conditions other than CVDs that are eligible for disease-specific rehabilitation coverage under the Japanese health insurance system

*DR* dysphagia rehabilitation, *NDR* no dysphagia rehabilitation, *CKD* chronic kidney disease, *MWST* Modified Water Swallow Test, *FT* Food Test *VE* videoendoscopic evaluation of swallowing, *VF* videofluoroscopic examination of swallowing

When comparing clinical backgrounds, the DR group was older and had a higher proportion of patients who had been in nursing facilities prior to hospitalization. In addition to common comorbid conditions, we also investigated the prevalence of chronic respiratory diseases, skeletal diseases, neurological diseases, disuse syndrome, and cancer, which we analyzed as rehabilitation-related comorbid conditions. These conditions are recognized for rehabilitation under the Japanese health insurance system, and their prevalence is considered important for assessing the severity of physical function decline in patients. As a result, cognitive disorders, neurological diseases, and cancer were more frequently observed in the DR group. These patients had longer hospital stays and higher in-hospital mortality rates.

Regarding the evaluation of swallowing function, the screening tests, including MWST and FT, were conducted, on average, 2.4 days after admission. Among the patients, 45.4% showed positive results in either of these tests. Additionally, 47 patients (11.1%) in the DR group required dental treatment during hospitalization.

Echocardiographic analysis, performed on 213 patients in the DR group and 269 in the NDR group, showed no significant difference in the EF between the groups (57.3% vs. 55.6%, *p* = 0.254), with the majority classified as having heart failure with preserved EF (70.0% vs. 68.0%). Plasma BNP levels at baseline were 687.2 ± 818.71 pg/mL in 308 DR patients and 816.2 ± 1121.6 pg/mL in 376 NDR patients (*p* = 0.093).

To clarify the clinical effects of integrating dysphagia rehabilitation during hospitalization, we analyzed the survivors with complete data on ADL scores (BI) at three points: pre-illness, admission, and discharge; we also analyzed the data on dietary intake and BMI at discharge. Of the 638 patients discharged alive, data necessary for the analysis were available for 314 patients, as shown in Table 2. A comparison of the in-hospital outcomes revealed a higher discharge rate to nursing homes in the DR group (Table 3). Despite the significantly higher number of patients requiring fasting or dietary restrictions in the DR group, the discharge oral caloric intake showed no difference between the groups. Similarly, no difference was noted in the daily caloric intake ratio calculated from the required basal metabolic rate using the Ganpule formula. BMI analysis showed that the DR group had lower minimum in-hospital BMI and pre-discharge BMI, with a greater prevalence of underweight patients (BMI < 18.5). However, the change in BMI from minimum in-hospital to pre-discharge showed no significant difference between the groups.

**Table 2:** Clinical background of surviving patients discharged with complete data on the BI and dietary intake

	DR group (*N* = 156)	NDR group (*N* = 158)	*p*-value
Age, years	86.4 ± 7.5	83.5 ± 8.0	< 0.01
Male	61 (39.1%)	67 (42.4%)	0.55
Underlying cardiac disease			
Ischemic heart disease	32 (20.5%)	35 (22.2%)	0.72
Valvular heart disease	29 (18.6%)	25 (15.8%)	0.52
Cardiomyopathy	5 (3.2%)	5 (3.2%)	0.98
Atrial fibrillation	66 (42.3%)	82 (51.9%)	0.09
Comorbidities			
Hypertension	94 (60.3%)	112 (70.9%)	0.05
Dyslipidemia	32 (20.5%)	54 (34.2%)	0.01
Diabetes mellitus	11 (7.1%)	16 (10.1%)	0.33
Hyperuricemia	20 (12.8%)	28 (17.7%)	0.23
CKD	33 (21.2%)	33 (20.9%)	0.95
Cognitive disorder	37 (23.7%)	27 (17.1%)	0.15
Rehabilitation-related Comorbid Conditions			
Chronic respiratory disease	23 (14.7%)	29 (18.4%)	0.39
Skeletal disease	24 (15.4%)	30 (19.0%)	0.40
Neurological disease	92 (59.0%)	70 (44.3%)	0.01
Disuse syndrome	75 (48.1%)	79 (50.0%)	0.73
Cancer	16 (10.3%)	11 (7.0%)	0.30
Residence before admission			
Own home	103 (66.0%)	104 (65.8%)	0.01
Nursing home	17 (10.9%)	5 (3.2%)	
Hospital (patient transfer)	36 (23.1%)	49 (31.0%)	
Length of hospital stay, days	46.7 ± 26.5	42.6 ± 27.3	0.19
≥ 30 days	106 (67.9%)	95 (60.1%)	0.15
Assessment of Swallowing Function			
Days from admission to assessment	2.2 ± 4.9		
Positive MWST (%)	53 (34.0%)		
Positive FT (%)	25 (16.0%)		
Positive MWST and FT (%)	21 (13.5%)		
Positive MWST or FT (%)	57 (36.5%)		
VE performed (%)	16 (10.3%)		
VF performed (%)	7 (4.5%)		
Implementation of dysphagia rehabilitation			
Number of days	16.5 ± 19.6		
Average daily time, min/day	36.8 ± 12.1		
Implementation of physical rehabilitation			
Number of days	33.1 ± 22.4	34.8 ± 26.2	0.55
Average daily time, min/day	68.4 ± 27.2	80.5 ± 27.2	< 0.01

Variables were reported as mean ± standard deviation for continuous variables and as frequencies (percentages) for categorical variables. The table represents the clinical background of surviving discharged cases

*BI* Barthel Index, *DR* dysphagia rehabilitation, *NDR* no dysphagia rehabilitation, *CKD* chronic kidney disease, *MWST* Modified Water Swallow Test, *FT* Food Test, *VE* videoendoscopic evaluation of swallowing, *VF* videofluoroscopic examination of swallowing

**Table 3:** Comparison of in-hospital outcomes between DR and NDR groups

	DR group (*N* = 156)	NDR group (*N* = 158)	*p*-value
Residence after discharge			
Own home	112 (71.8%)	134 (84.8%)	0.02
Nursing home	36 (23.1%)	20 (12.7%)	
Hospital (patient transfer)	8 (5.1%)	4 (2.5%)	
Patients requiring fasting	69 (44.2%)	28 (17.7%)	< 0.01
*Fasting Period, days	3.8 ± 8.3	1.8 ± 2.0	0.21
Energy intake at discharge, kcal/day	1159.8 ± 428.7	1232.0 ± 380.7	0.12
†Estimated BMR, kcal/day	722.7 ± 215.0	785.7 ± 218.8	0.01
EI:BMR ratio			
< 1.0	33 (21.2%)	32 (20.3%)	0.88
1.0–1.39	53 (34.0%)	58 (36.7%)	
≥ 1.4	70 (44.9%)	68 (43.0%)	
Minimum BMI during hospitalization	19.4 ± 3.7	20.5 ± 4.0	0.01
BMI at discharge	20.0 ± 3.7	21.0 ± 3.9	0.02
ΔBMI (Discharge – Minimum)	0.6 ± 1.2	0.5 ± 1.2	0.79
BMI ≥ 18.5	91 (58.3%)	118 (74.7%)	< 0.01
ADL score (BI) at discharge	61.6 ± 37.2	81.3 ± 25.6	< 0.01
Mobility score ≥ 10	96 (61.5%)	133 (84.2%)	< 0.01
Toilet use score = 10	87 (55.8%)	119 (75.3%)	< 0.01
ΔBI (Discharge – Admission)	15.9 ± 30.9	15.0 ± 27.7	0.79
ΔBI (Discharge – Pre-illness)	-3.5 ± 25.3	0.5 ± 23.6	0.15

Variables were reported as mean ± standard deviation for continuous variables and as frequencies (percentages) for categorical variables. The table represents the outcomes associated with rehabilitation. *The fasting period indicates the number of days patients who required fasting or dietary restrictions from hospital admission to the initiation of oral feeding. ^†^Estimated basal metabolic rate was estimated using Ganpule’s formula, providing insight into metabolic requirements at the point of discharge. ^‡^The mean days for recording the lowest BMI was 23.3 ± 20.9 for the NDR group and 26.5 ± 23.5 for the DR group (*p* = 0.213)

*BI* Barthel Index, *DR* dysphagia rehabilitation, *NDR* no dysphagia rehabilitation, *BMR* basal metabolic rate, *EI* energy intake at discharge, *BMI* body mass index, *ADL* activities of daily living, ΔBI change in the Barthel Index score, where a positive value indicates improvement

**Fig. 2:**
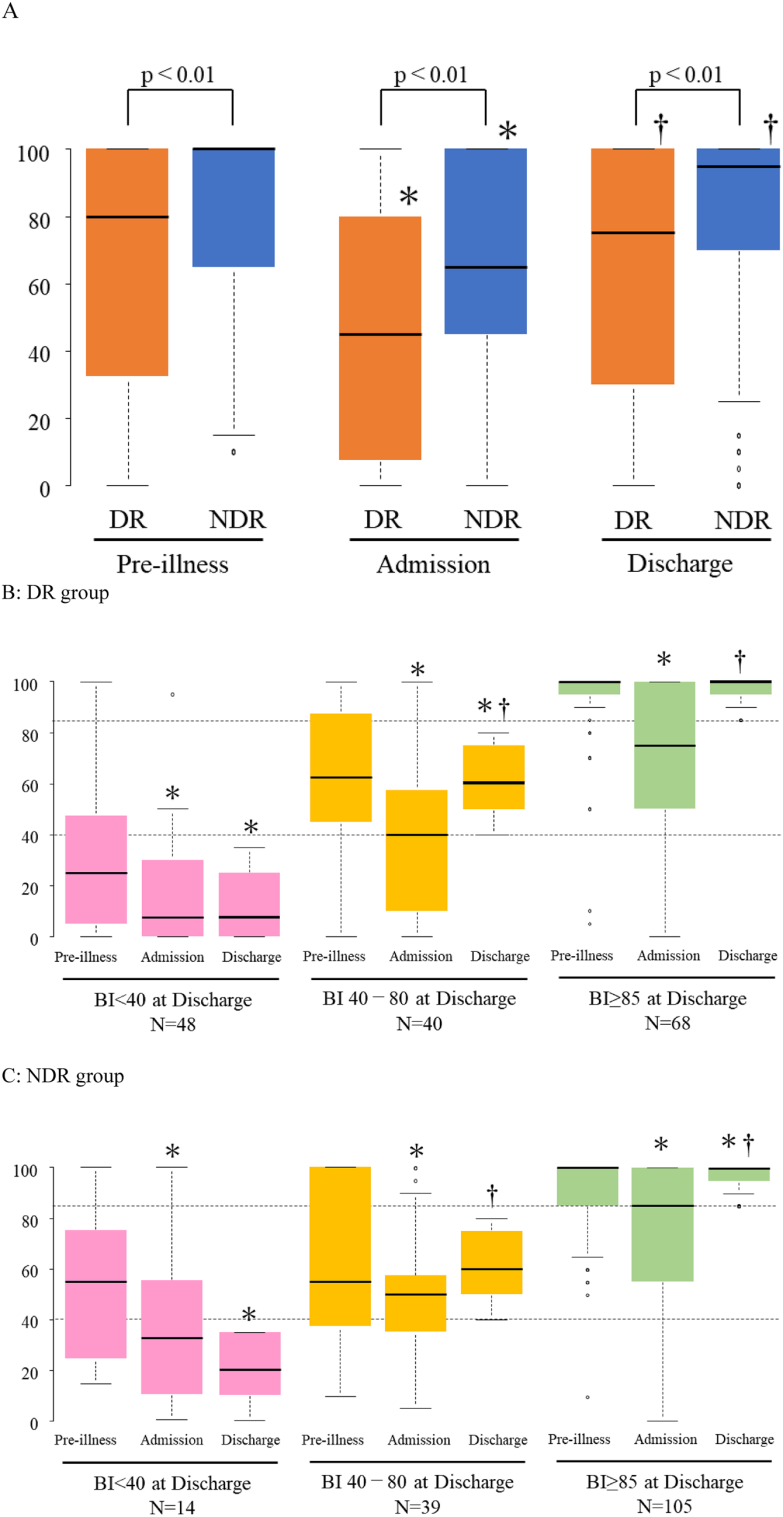
Changes in the BI scores across different time points in surviving patients at discharge. The box-and-whisker plot illustrates the median BI scores of surviving patients pre-illness, at admission, and at discharge. The boxes represent IQRs, while the whiskers extend to 1.5 times the IQR. Outliers are represented as individual dots. The asterisks (*) denote statistically significant differences compared to the pre-illness scores, and the daggers (†) indicate significant differences compared to the admission scores (p < 0.05). **A** the temporal changes in the ADL scores in the DR and NDR groups illustrate the ADL trajectories from pre-illness to discharge, highlighting the comparative outcomes between the two groups. The BI for the DR group, the values were 80 [33.8–100], 45 [8.8–80], and 75 [30–100], respectively. For the NDR group at pre-illness, admission, and discharge were 100 [65–100], 65 [45–100], and 95 [70–100], respectively. **B** and **C** the temporal changes in the ADL scores stratified by discharge BI category for the DR and NDR groups, respectively. Each figure categorizes patients into three BI ranges (< 40, 40–80, and ≥ 85), demonstrating the progression of ADL scores from admission to discharge within these categories. **B** in the DR group, the BI at pre-illness, admission, and discharge for the BI < 40 at discharge subgroup were 25 [5–46.3], 7.5 [0–30], and 7.5 [0–25], respectively. For the BI 40–80 at discharge subgroup, the values were 62.5 [45–86.3], 40 [10–56.3], and 60 [50–75], respectively. For the BI ≥ 85 at discharge subgroup, the values were 100 [95–100], 75 [50–100], and 100 [95–100], respectively. **C** In the NDR group, the BI at pre-illness, admission, and discharge for the BI < 40 at discharge subgroup were 55 [28–73.8], 32.5 [10–55], and 20 [10–33.8], respectively. For the BI 40–80 at discharge subgroup, the values were 55 [38–100], 50 [35–57.5], and 60 [50–75], respectively. For the BI ≥ 85 at discharge subgroup, the values were 100 [85–100], 85 [55–100], and 100 [95–100], respectively. *BI* Barthel Index, *IQR* interquartile ranges, *ADL* activities of daily living, *NDR* no dysphagia rehabilitation, *DR* dysphagia rehabilitation

**Fig. 3:**
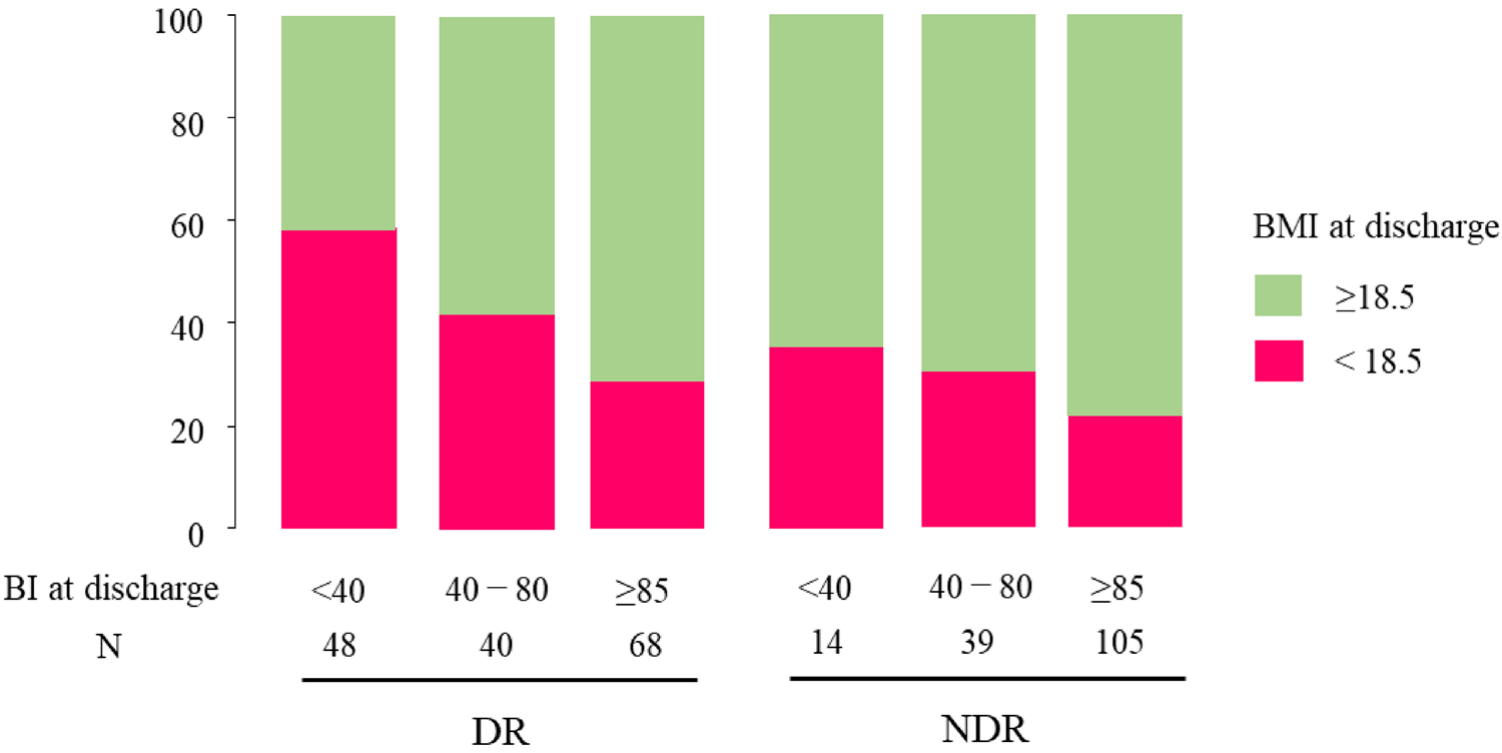
Proportion of patients with a discharge BMI of ≥ 18.5, categorized by their discharge BI values for the DR and NDR groups. Statistical analysis revealed no significant difference in the NDR group across BI categories (*p* = 0.357), whereas significant differences were found in the DR group (*p* < 0.01), which indicates variability in the BMI outcomes related to the severity of disability as measured by the BI. *BMI* body mass index, *BI* Barthel Index, *DR* dysphagia rehabilitation, *NDR* no dysphagia rehabilitation

To further understand the impact on patients’ daily functioning, we analyzed the temporal changes in ADL. The temporal changes in ADL demonstrated consistently lower ADL levels in the DR group than in the NDR group during the pre-illness, admission, and discharge phases (Fig. 2A). However, the degree of recovery from admission to discharge was comparable between the groups (Table 3). The analysis was stratified by the BI scores of the patients at discharge and categorized into three levels: < 40, 40–80, and ≥ 85 (Fig. 2B, C). For both the DR and NDR groups, patients with a discharge BI of ≥ 40 exhibited significant improvements in ADL from admission to discharge. Conversely, patients with a discharge BI of < 40 did not show any significant improvement. Furthermore, we examined the proportion of patients with a BMI of ≥ 18.5 at discharge, categorized by their ADL status using the BI at discharge in three levels: < 40, 40–80, and ≥ 85 (Fig. 3). In the NDR group, a greater proportion of patients across all BI categories had a BMI of ≥ 18.5. Notably, within the DR group, patients with higher discharge BI scores were more likely to have a BMI of 18.5 or higher. Additionally, we conducted an analysis to identify factors influencing the lack of ADL independence at discharge.

Logistic regression analysis focusing on factors influencing the lack of ADL independence at discharge (BI < 85) identified the need for dysphagia rehabilitation as a strong independent predictor (odds ratio, 2.359), independent of age, sex, length of hospital stay, pre-illness ADL, presence of dementia, and the number of diagnoses eligible for rehabilitation (Table 4). It is noteworthy that while the number of rehabilitation-related comorbid conditions was not a significant factor, the need for dysphagia rehabilitation showed a strong association with lack of ADL independence at discharge. This association remained significant even after excluding patients who were presumed to be completely dependent before their illness (BI < 40).

**Table 4:** Binary logistic regression analysis for risk factors associated with ADL dependence at discharge (BI < 85)

	Model 1: All cases included (*N* = 432)			Model 2: Cases with pre-illness BI ≥ 40 (*N* = 348)		
	OR	95% CI	*p*-value	OR	95% CI	*p*-value
Age (per 1 year)	1.058	1.02–1.098	< 0.01	1.048	1.009–1.089	0.02
Male (yes vs. no)	0.995	0.589–1.681	0.99	0.973	0.564–1.679	0.92
Hospital stay (per 7 days)	1.116	1.038–1.2	< 0.01	1.132	1.05–1.221	< 0.01
Pre-illness ADL (BI, per 5 points)	0.948	0.936–0.96	< 0.01	0.942	0.928–0.957	< 0.01
Cognitive disorder (yes vs. no)	1.513	0.779–2.941	0.22	1.355	0.672–2.735	0.40
Number of rehabilitation-related comorbidities	1.229	0.908–1.662	0.18	1.175	0.857–1.61	0.32
Need for dysphagia rehabilitation (yes vs. no)	2.359	1.4–3.976	< 0.01	2.576	1.489–4.456	< 0.01

*ADL* activities of daily living, *BI* Barthel Index, *OR* odds ratio, *CI* confidence interval

## Discussion

This study elucidated the current status of dysphagia in older patients hospitalized for CVD, highlighting the role of in-hospital dysphagia rehabilitation interventions in improving oral intake, BMI, and ADLs. The finding that the need for dysphagia rehabilitation was a strong predictor of ADL independence emphasizes the critical perspective that active engagement in dysphagia management can significantly improve the QOL in older patients with CVD.

In community hospitals focusing on patients aged ≥ 65 years, more than half of the patients with CVD required dysphagia rehabilitation. These patients were older and had a higher prevalence of comorbidities, such as cognitive disorders, neurological diseases, and cancer, suggesting a relationship between these conditions and the causes of dysphagia. Previous reports have indicated that dysphagia is present in 23–40% of patients with heart failure, highlighting an increase in prevalence with aging [2, 3, 8, 9]. Although certain cardiovascular conditions have been reported to directly cause dysphagia through mechanisms such as left atrial enlargement or external compression of the esophagus by the aneurysmal thoracic aorta [10, 11] and congestion leading to syndromes resembling normal pressure hydrocephalus [12], these are considered rare occurrences. Common comorbidities in aging patients, such as cognitive impairment, neurological disorders, and nutritional problems, are thought to be strongly associated with frailty [2].

While we were unable to assess the severity of dementia or cerebrovascular disorders because of the retrospective nature of our study, our results showed that despite the need for dysphagia rehabilitation, the positive dysphagia screening rate was 45.4%, suggesting that factors other than direct swallowing disorders, such as cognitive impairment and physical functional decline, are significant contributors. Therefore, dysphagia in older patients with CVD may be attributed not solely to the cardiovascular conditions themselves but also due to the presence of comorbidities and age-related factors.

The coexistence of dysphagia and its contributing factors, such as frailty, is likely to complicate the management of older patients, highlighting the need for comprehensive rehabilitation that addresses both physical and cognitive aspects. In addition, the presence of dysphagia prolongs hospital stay and increases social, medical, and caregiver burden [8]. The higher rate of discharge to nursing homes in the DR group suggests that dysphagia has a significant impact on the post-discharge living environments of older patients. Dysphagia has been identified as a major barrier to home discharge in hospitalized patients with heart failure [9] and is associated with a decline in physical function during cardiac rehabilitation [13], as well as with functional recovery and mortality over 1 year [3].

Regarding the outcomes of dysphagia rehabilitation during hospitalization, despite the longer fasting period in the DR group, no difference was observed in oral caloric intake at discharge between the groups. The ratio of actual oral intake to basal metabolic rate was comparable between the DR and NDR groups, and only over 40% of the patients exceeded 1.4 times their basal metabolic rate, which is considered the threshold for mild activity levels [14]. In the DR group, although the absolute values were lower, no difference was observed in the degree of BMI or ADL improvement compared with the NDR group. However, this interpretation must be tempered by the fact that the study design did not allow for a clear delineation of the effect of dysphagia rehabilitation alone. Among the patients who were discharged alive, the improvement in caloric intake and the similar degree of BMI and ADL improvement between the groups suggested that dysphagia rehabilitation is a critically important intervention for the recovery of older patients. In contrast, patients who required full assistance at discharge (BI < 40) had low ADL scores before admission, suggesting that comprehensive interventions, including dysphagia rehabilitation, did not lead to improvements. This outcome, supports shared decision-making for these patients, highlighting the need for realistic expectations and tailored treatment plans. Furthermore, within the DR group, a high discharge ADL score was associated with a low proportion of patients with a BMI < 18.5, indicating that effective dysphagia rehabilitation can contribute to improvements in both ADL and BMI. Although previous studies have shown these associations, to our knowledge, this is the first study to demonstrate the interventional effect of dysphagia rehabilitation. Owing to the absence of pre-illness body composition data, assumptions had to be made regarding the baseline BMI of the DR group, leading to the speculation that this group had a higher prevalence of patients with low pre-illness BMI.

The significance of dysphagia assessment and intervention must be noted. The need and effectiveness of dysphagia rehabilitation in older hospitalized patients with CVD support the need for incorporating dysphagia management into the comprehensive care of these patients. Although nutritional status assessment and counseling are mentioned in many guidelines and reviews on heart failure and cardiac rehabilitation, assessments and interventions for dysphagia function are not addressed [6, 15–17]. Our research findings and previous reports indicate that dysphagia is common in older patients with heart failure and is a significant issue affecting life prognosis and QOL as well as rehabilitation outcomes, discharge destinations, and all-cause mortality. Early identification and treatment of dysphagia in this patient population is essential for improving health outcomes and QOL.

### Limitations and future directions

Although this study provides valuable insights into the benefits of dysphagia rehabilitation, it has some limitations. The retrospective design limits the ability to establish causality, and the single-center design may have affected the generalizability of the findings. Since the study included a very old population, the number of subjects included in the outcome analysis was significantly reduced because of missing data. Future studies should aim to conduct prospective multicenter trials to validate these findings and investigate the long-term effects of dysphagia rehabilitation on patient outcomes. Further research should be conducted on the specific components of dysphagia rehabilitation programs that are effective in improving ADL independence and nutritional status. Exploring the role of technology and innovative approaches in dysphagia management may also provide new directions for improving patient care.

## Conclusion

This study highlights the high prevalence of dysphagia among older patients with CVD and the significant benefits of dysphagia rehabilitation in enhancing ADL independence. Integrating dysphagia management into the comprehensive care of older patients with CVD is crucial, considering its positive impact on oral intake, nutritional status, and overall QOL. Despite the limitations of this study, these findings advocate the adoption of multidisciplinary strategies that focus on the early identification and timely intervention for dysphagia, aimed at enhancing recovery and minimizing healthcare burdens. Future research should aim to identify the most effective elements of dysphagia rehabilitation and explore innovative approaches to improve care for this population.

## Data Availability

The data underlying this article cannot be shared publicly owing to privacy or ethical restrictions. For more information regarding the data and access requests, please contact the corresponding author.
